# Carbohydrate resources drive increased ant recruitment in a saline coastal ecosystem

**DOI:** 10.1007/s00114-026-02136-w

**Published:** 2026-07-20

**Authors:** Lucas Gütler, Jefferson Bruno Bretas de Souza Oliveira, Tathiana Guerra Sobrinho, Vanessa Soares Ribeiro, Lucas Navarro Paolucci

**Affiliations:** 1 Secretaria Municipal de Desenvolvimento Urbano e Meio Ambiente, Prefeitura Municipal de Colatina, Colatina, Brazil; 2 Departamento de Biologia Geral, Centro de Ciências Biológicas e da Saúde, Laboratório de Interações, Ecológicas e Biodiversidade, Universidade Estadual de Montes Claros, Montes Claros, MG Brazil; 3 Departamento de Ciências Agrárias e Biológicas, Universidade Federal do Espírito Santo, São Mateus, Brazil; 4 Instituto de Pesquisa Ambiental da Amazônia, Brasília, Distrito Federal, Brazil; 5 Programa de Pós-Graduação em Ecologia, Departamento de Biologia Geral, Universidade Federal de Viçosa, Viçosa, MG Brazil; 6 Departamento de Biologia Geral, Universidade Federal de Viçosa, Viçosa, MG Brazil

**Keywords:** Behavioral ecology, Coastal ecosystems, Nutritional ecology, Nutrient regulation, Resource limitation

## Abstract

Maintaining a physiological stoichiometric balance is a fundamental driver of foraging behaviour; yet, resource availability varies across trophic levels and vegetation strata. Therefore, primary consumers and predators may experience distinct nutritional opportunities between epigaeic and arboreal habitats. We investigated how ant communities respond to carbohydrates availability in the Brazilian Restinga – a coastal ecosystem characterized by sodium saturation and low carbohydrate availability. For that, we experimentally supplied carbohydrates (C₁₂H₂₂O₁₁, 20%), salt (NaCl, 1%) and water in both epigeic and arboreal strata to assess recruitment responses across the overall ant community as well as predominantly primary consumers and predominantly predatory ants separately. Carbohydrates were more attractive to the overall ant community, attracting eight times more species and 15 times more ant individuals than salt. This higher recruitment was consistent across feeding guilds, with primary consumers and predators exhibiting 17-fold and 42-fold increases in individual recruitment to carbohydrates as many as salt baits. Although primary consumers were recruited more in the arboreal stratum and predators in the epigeic stratum, recruitment to carbohydrate baits was consistent across vegetation strata for both the overall ant community and the feeding guilds. In conclusion, our findings show that carbohydrates in a saline coastal ecosystem strongly act as an important resource influencing ants’ foraging behaviour across vegetation strata. These results highlight how stoichiometric imbalances dictate foraging decisions and community dynamics and provide insights into how resource availability influences ant-mediated ecological functions in saline environments.

**Supplementary Information:**

The online version contains supplementary material available at 10.1007/s00114-026-02136-w.

## Introduction

Foraging is the overall process that encompasses several mechanisms by which organisms acquire resources needed for growth, survival, and reproduction, thereby directly determining their performance and fitness in natural habitats (Tilman 1982; Raubenheimer et al. 2009). Under nutrient-limited conditions across several habitats and trophic groups within a given ecosystem, organisms may concentrate their foraging efforts on acquiring the scarcest nutrients to maintain the physiological stoichiometric balance required for organismal fitness (Rothman et al. 2006; Reynolds et al. 2009; Lasmar et al. 2023). Therefore, the availability of key nutrients, such as carbohydrates and sodium, can shape the foraging behavior and success of organisms and may contribute to structuring biological communities (Sterner and Elser 2002; Simpson et al. 2006). Therefore, by influencing organisms’ foraging for different resource types, nutrient availability can modulate key ecosystem functions — such as predation, scavenging, seed dispersal, and nutrient cycling — through ecological interactions (Kaspari and Yanoviak 2009; Tilman et al. 2014). Understanding how nutrient availability shapes the organism’s foraging behavior is therefore essential for studies of ecological processes that maintain ecosystem functioning and health (Requier et al. 2019).

The spatial distribution of nutrients within ecosystems, influenced by biotic and abiotic factors, can shape nutritional requirements across habitats and trophic groups and direct foraging efforts towards essential resources for growth and metabolism (Stephens and Krebs 2019). In nutrient-dependent ecosystems, nutrient availability often varies spatially - both horizontally and vertically - creating distinct nutritional landscapes that influence animal activity (Law and Parr 2019). For instance, in many terrestrial ecosystems, ground-level resources — primarily derived from animal organic matter — are enriched with sodium (Clay et al. 2015; Kaspari et al. 2020), whereas arboreal resources of plant origin are typically carbohydrate-rich (Davidson et al. 2003; Gibb and Cunningham 2009; Houle et al. 2014). This vertical stratification likely influences the foraging preferences of different trophic groups, ultimately affecting community structure and species functions by altering resource use, habitat occupancy, and trophic interactions (Fowler et al. 2014; Law and Parr 2019).

Ants serve as ideal ecological models for studying foraging behavior and nutritional requirements due to their high diversity, abundance, and central roles in ecosystem processes (Tiede et al. 2017; Lasmar et al. 2023). Ants occupy different trophic groups across vegetation strata (Yanoviak and Kaspari 2000), ranging from predominantly primary consumers of plant exudates to predominantly predators (Blüthgen and Feldhaar 2010; Griffiths et al. 2018). Because these groups differ in nutritional demands, their foraging behavior often mirrors the specific nutrient availability of their habitats (Kaspari and Yanoviak 2001; Ness et al. 2009). Among essential nutrients for ants, carbohydrates and sodium are critical; carbohydrate-rich resources enhance thermal tolerance and immunity (Kay et al. 2014; Bujan and Kaspari 2017), while sodium is vital for osmoregulation, muscle contraction and neural activity (Da Silva and Williams 2001). Given that these nutrients are distributed unevenly across resources, different trophic and vertical groups face distinct constraints. For instance, predominantly primary consumer ants (species that feed predominantly on plant-derived resources, including extrafloral nectar and honeydew) are typically limited by sodium and protein (Davidson 1997; Kaspari 2020), whereas predominantly predatory ants are often limited by lipids and carbohydrates (Kaspari and Yanoviak 2001; Wilder et al. 2013). Imbalances in nutritional distribution are also evident across vertical groups: arboreal ants, which feed on plant exudates rich in carbohydrates, are sodium-limited (Blüthgen et al. 2004; Rico-Gray and Oliveira 2007; Nepi et al. 2012), while epigeic ants are more exposed to protein and sodium but are carbohydrate-limited (Kaspari et al. 2012).

The Brazilian Restinga, which are coastal sandy plain ecosystems associated with the Brazilian Atlantic Forest domain, are characterized by high salinity and sodium saturation, particularly in the soil (Araújo and Lacerda 1987; Scarano 2002; Maun 2009). Conversely, this ecosystem is carbohydrate-poor, as this resource is most available in the arboreal strata, and only about 13% of plants have extrafloral nectaries (e.g., Miranda et al. 2022). This environment contrasts sharply with inland habitats, where carbohydrate-based resources are abundant (e.g., Oliveira and Brandão 1991; Oliveira et al. 2020; Cavalcante et al. 2024) but sodium is scarce, driving a strong foraging preference for this nutrient (Lasmar et al. 2021, 2023). For instance, ants are strongly attracted to sodium in inland ecosystems, but they are much less attracted to salty baits in saturated-salt locations (Kaspari et al. 2008, 2010). However, a significant knowledge gap remains regarding how carbohydrate availability shapes foraging preferences in these salt-saturated environments. Carbohydrate availability may affect ant trophic groups differently in coastal ecosystems, as predominantly primary consumer ants are predominantly arboreal, whereas predominantly predatory ants are mainly epigeic (i.e., forage on the soil surface).c Understanding these responses is essential for identifying the nutritional constraints that shape ant foraging in coastal ecosystems.

We used ants as a model group to investigate nutrient requirements in highly saline environments, where nutrients other than sodium – especially carbohydrate – may be limited. Through a field experiment conducted in the Brazilian Restinga, we assessed ants’ foraging behavior across vertical vegetation strata (ground and arboreal), focusing on two essential nutrients for ant fitness (sodium and carbohydrate). Because organisms are expected to strongly recruit on limiting resources regardless of trophic position, we asked: how does the availability of scarce and saturated nutrients influence ants’ responses across trophic groups and vegetation strata in a highly saline environment? To address this question, we tested three hypotheses: (i) the overall ant community, as well as predominantly primary consumer and predominantly predatory ants separately, recruit more strongly to carbohydrate than to sodium, regardless of vegetation stratum; (ii) predominantly primary consumer ants forage more strongly in the arboreal stratum, regardless of their resource-type preferences; and (iii) predominantly predatory ants forage more strongly in the epigeic stratum, regardless of their resource-type preferences. Based on these hypotheses, we expect higher ant species richness and more ant recruitment to scarce resources (carbohydrates) than to saturated ones (sodium) across both vegetation strata, irrespective of the habitat preferences of different trophic groups.

## Materials and methods

### Study area

We conducted this study in the Parque Estadual de Itaúnas (18º20’/18º25’S − 39º40’/39º42’W), a protected coastal area located in the north of Espírito Santo state, Brazil, covering an area of 3,481 hectares (CEPEMAR 2004). The region is predominantly covered by Restinga vegetation, a foodable highly saline coastal ecosystem characterized by sandy formations running parallel to the shoreline, encompassing beaches, dunes, and coastal plains under strong marine influence (IEMA 2005). Its vegetation ranges from herbaceous communities to low-stature forests on average 12 m in height — with a few plant species exhibiting extrafloral nectaries — including shrubby and arboreal formations acting as a natural barrier against coastal erosion and storm surges (Miranda et al. 2022; Pereira and Menezes 2023). This heterogeneous mosaic changes with increasing distance from the ocean as salt influence gradually decreases. The ecosystem is characterized by nutrient-poor soils, strong winds, high solar radiation, and limited availability of surface freshwater (Araújo and Lacerda 1987; Maun 2009; Souza et al. 2016). The park is located in a region with an Aw climate - seasonal tropical according to the Köppen classification, with an average annual rainfall of 1308 mm and a relative humidity of 83%. It has an average annual temperature ranging from 21.7 °C to 26.7 °C, with rainfall concentrated during the summer (from December to March) (CEPEMAR 2004).

## Sampling design

**Fig. 1:**
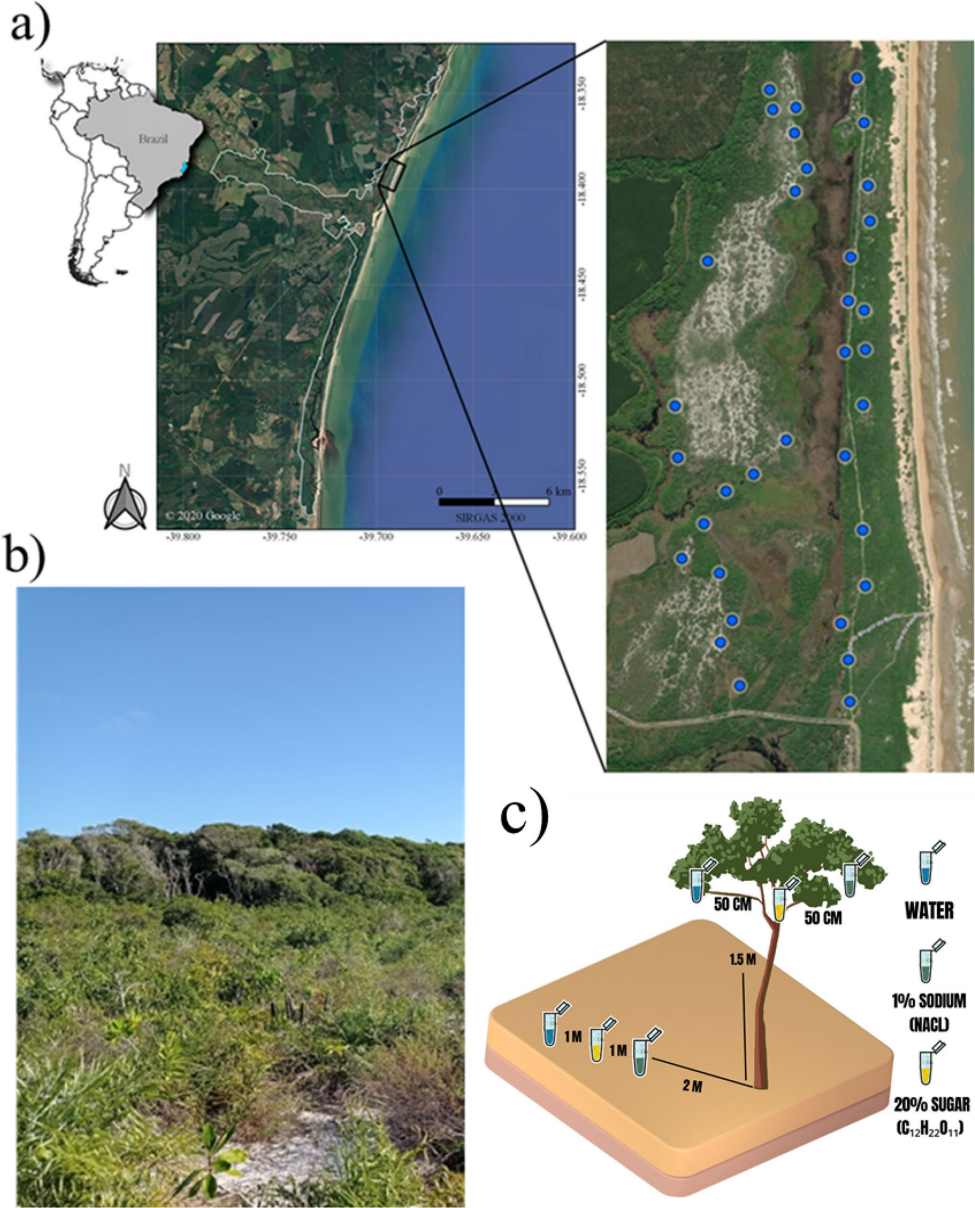
**a**) Delimitation of the State of Espírito Santo in Brazil (Adapted from Miranda et al. 2022), highlighting the delimitations of the Parque de Itaúnas-ES - white line, showing the 28 sampling points in blue. Images from Google Earth; **b**) Image of the study area, highlighting the variation in the existing Restinga vegetation types in the location (original photograph); **c**) A schematic representation of the sampling stations in the epigeic and arboreal strata

We established 28 sampling points in our study area (Fig. 1a). Each sampling point was defined by the presence of the plant species *Pera glabrata* (Schott) Poepp. ex Baill (Peraceae). This species can reach up to 20 m in height, lacks extrafloral nectaries, and is abundant and widely distributed in the Restinga we studied (Freitas et al. 2011). The choice of *Pera glabrata* was intended to avoid introducing local variation associated with the presence of extrafloral nectaries, which can represent an important carbohydrate resource for many ant species. By selecting a tree species without extrafloral nectaries, we standardized one potential source of spatial variation in ant activity among sampling points, while recognizing that ants exploit multiple carbohydrate resources in natural environments. Sampling points were spaced at least 200 m apart, and distributed over an area of approximately 2 km², with a distance of up to 900 m from the sea. Although the points were not arranged strictly parallel to the coastline, they were all located within the same coastal physiognomy, minimizing large differences in natural sodium availability among sampling sites.

We conducted foraging experiments in June 2023. In each sampled plant, we established two sampling stations: one at ground level (epigeic strata) and the other on the tree (arboreal strata) (Fig. 1c). In each station, we placed three nutritional treatments in 1.5 mL Eppendorf tubes, as done in previous studies (e.g., Guariento et al., 2021; Lasmar et al. 2021). Each tube contained one of the different nutritional resources dissolved in water and embedded in cotton: 1% sodium (NaCl), 20% sugar (carbohydrates) (C₁₂H₂₂O₁₁), and water as a control (following Lasmar et al. 2021, 2023). In the epigeic stratum, Eppendorf tubes were positioned 2 m from the selected tree, spaced 1 m apart. In the arboreal stratum, the tubes were tied to branches or trunks with wires at a height of 1.5 m, with 0.5 m between them. The experiment was conducted between 7:00 AM and 11:00 AM to align with the ants’ active foraging period in the study area (Guimarães et al. 2023). The tubes were left open for 2 h, then capped to collect the ants present inside and on the surface.

Captured ants were stored in 70% alcohol and taken to the laboratory for sorting and identification. We identified all specimens to the lowest possible taxonomic level following Baccaro et al. (2015), and the identifications were confirmed by a specialist. We categorized all ants into two trophic groups (primary consumers and predators) as in Lasmar (2022). Ant morphospecies were classified using the guide to ant genera of Brazil (Baccaro et al. 2015), based on knowledge about the genus. Furthermore, we also used field observations for categorization, such as for the species *Ectatomma tuberculatum*, which is recognized as a predator (Ribeiro et al. 2019), but in the study area is mostly found associated with NEFs, indicating that in this environment, this species exhibits a feeding habit that diverges from what is already established in the literature. This approach ensures that species identified only to the genus are correctly classified into trophic groups. Although such categorization is limited due to ants’ dietary plasticity, classifying species according to their predominant feeding habits provides a useful framework for detecting broad ecological patterns at the community level. This approach has been successfully used across different environments (subterranean, epigaeic, and arboreal) and Brazilian biomes (Lasmar 2022; Lasmar et al. 2023).

## Data analyses

We used two complementary approaches to evaluate how resource availability influences organisms' foraging behavior across vegetation strata according to nutritional requirements under resource-limited conditions. First, we developed statistical models based on the recruitment activity of the overall ant community (hereafter, “community level”). Specifically, we assessed how all observed ant groups collectively responded to resource availability. For this approach, we used the total number of ants (recruitment) recorded in tubes containing each resource type (water, sugar, and salt) across vegetation strata (arboreal and epigaeic) as a proxy for foraging effort (Kaspari et al. 2019). Additionally, we used ant species richness foraging on each resource type across strata as a complementary measure of community-level foraging effort. In a second approach, we divided the overall ant community into guilds of predominantly primary consumers and predominantly predators (see *Ant Foraging Sampling*) and evaluated the total number of ants belonging to each feeding guild (i.e., foraging effort) recorded in tubes containing each resource type within each vegetation stratum.

For these approaches, we constructed four generalized linear mixed models (GLMMs). Two models were developed at the community level, using (i) the total number of individuals and (ii) ant species richness as response variables. In addition, two guild-level models were constructed, one for predominantly primary consumers and another for predominantly predators. In each guild-level model, the total number of individuals belonging to the respective guild was used as the response variable. In all models, resource type and vegetation stratum were included as categorical explanatory variables, along with their interaction. Because resources provided across strata within each sampling point were placed beneath the same plant, the sampling point was included as a random effect in all models to account for the non-independence of observations between strata. All analyses were conducted in R (R Core Team 2024) using the *glmmTMB* package (Brooks et al. 2025). For each model, we initially fitted a Poisson distribution, which is appropriate for count data. Model assumptions were evaluated using simulated residuals generated with the *DHARMa* package. Residual diagnostics included tests for uniformity (Kolmogorov–Smirnov test), dispersion, outliers, and homogeneity of variance among groups. Model fit was considered adequate when no significant deviations from the expected residual distribution were detected (Hartig 2022).

When model diagnostics indicated violations of assumptions, we fitted alternative models incorporating adjustments for subdispersion, zero inflation or heterogeneous dispersion. Accordingly, we evaluated negative binomial (nbinom1), zero-inflated (*ziformula*), and heterogeneous dispersion (*dispformula*) models. The *ziformula* component estimates the probability of structural zeros, i.e., zeros that cannot be adequately explained by the Poisson or negative binomial count process alone. This component is fitted as an additional binomial model with a logit link function, allowing the probability of a structural zero to vary as a function of explanatory variables while remaining bounded between 0 and 1. In contrast, the *dispformula* allows the dispersion parameter to vary as a function of explanatory variables rather than assuming a constant level of dispersion across all observations. This parameter is modeled using a logarithmic link function, ensuring positive estimates and allowing the model to account for heteroscedasticity (i.e., unequal variance) among groups (see Brooks et al. 2017, 2025 for details).

Different models (poisson, nbinom1, poisson or nbinom1 + *ziformula*, and poisson or nbinom1 + *dispformula*) were compared using Akaike’s Information Criterion (AIC) implemented in the *bbmle* package (Bolker and R Development Core Team 2023). The final model was selected based on both diagnostic performance and overall model fit. Through the best-fitting models, we evaluated the significance of explanatory effects using the Type III Anova function (because interactions between explanatory variables were initially included) implemented in the *car* package (Fox and Weisberg 2019). Following a model simplification procedure, in which the interaction between explanatory variables was removed, Type II ANOVA was applied. Post hoc comparisons were performed using the *emmeans* package whenever significant resource-type effects were detected, with Tukey-adjusted multiple comparisons (Lenth 2024).

## Results

We recorded 606 foraging ant individuals from 25 species across six subfamilies. Myrmicinae (445 individuals and 12 species) and Formicinae (101 individuals and 6 species) were the most representative subfamilies, comprising 73.43% and 16.60% of the sampled individuals, respectively. The community was characterized by high dominance from a few species: *Pheidole* sp. 2 (*n* = 134 individuals), *Wasmannia auropunctata* (*n* = 78), and *Cephalotes pusillus* (*n* = 76), which together comprised nearly half (47.5%) of the total ant abundance. (Tables S1–S4).

**Fig. 2:**
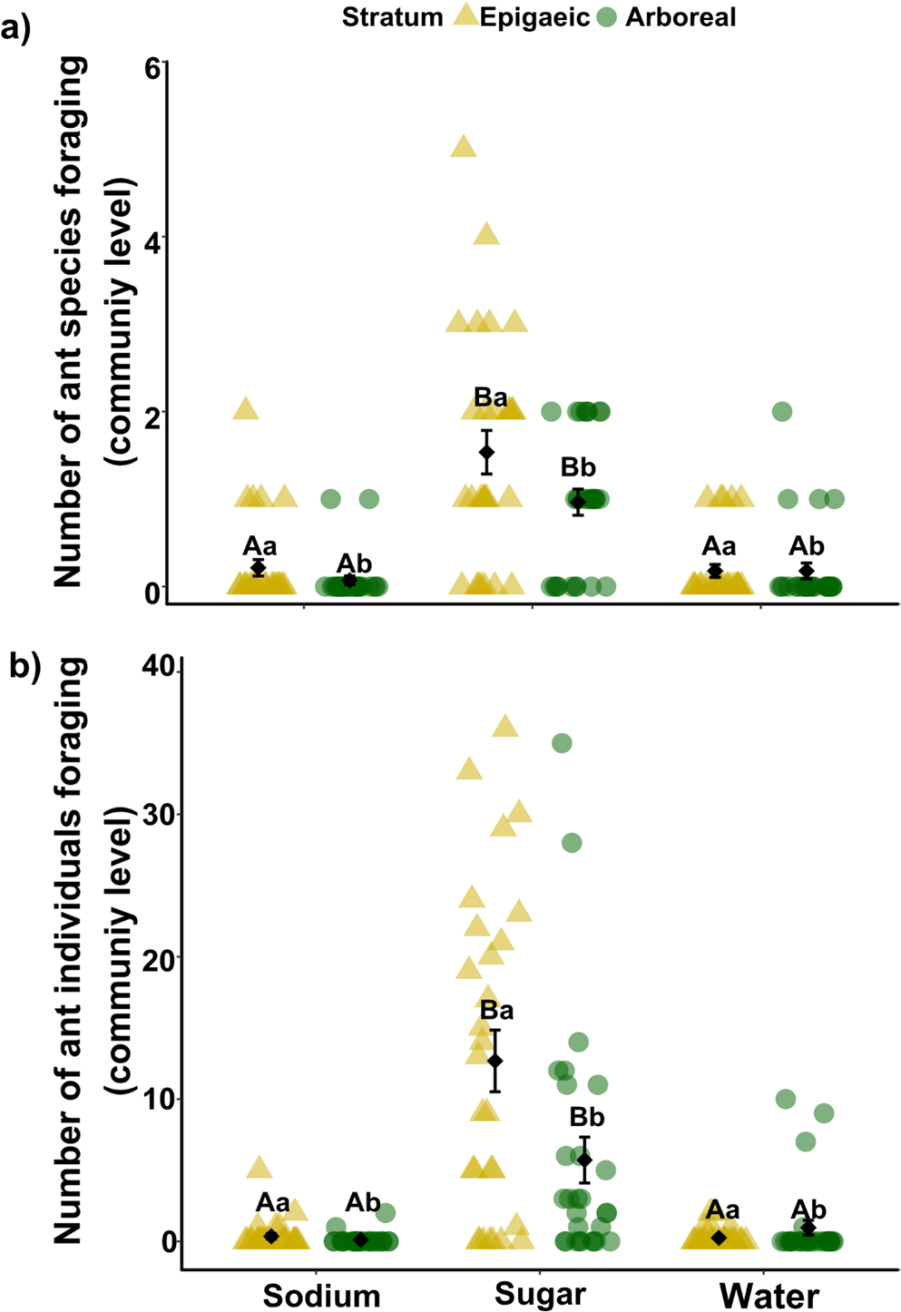
Mean and variation (SE) of the number of species **(a)** and the number of individuals **(b)** of the overall ant community foraging across strata (epigeic and arboreal) and different resources (sodium, sugar and water) within the floodable forest formation at a Brazilian Restinga (a coastal ecosystem highly saline and poor in sugar). Yellow triangles represent units in the epigeic stratum, while green circles represent units in the arboreal stratum. Black bars represent standard error, and black diamonds represent the means. Capital letters indicate post hoc comparisons among resource types, whereas small letters indicate comparisons between strata. Treatments sharing the same letter do not differ significantly, whereas different letters indicate significant differences (α = 0.05)

Of the 25 species identified, nine were classified as primary consumers (*n* = 202) and 16 as predators (*n* = 403). *C*. *pusillus* (*n* = 76 individuals), *Brachymyrmex* sp. 1 (*n* = 49), and *Azteca* sp. 1 (*n* = 35) were the most representative species of predominantly primary consumers, which together accounted for 79.2% of the individuals assigned to this guild. Predominantly predators were primarily represented by *Pheidole* sp. 2 (*n* = 134), *W*. *auropunctata* (*n* = 78), and Pheidole sp. 5 (*n* = 44), which together accounted for 63.4% of this guild. Ant attraction was heavily skewed toward carbohydrates: 22 species (*n* = 556) were recorded on sugar baits, compared to only six species each on sodium (*n* = 12) and water (*n* = 37) Sugar baits were dominated by *Pheidole* sp. 2 (*n* = 129), *W. auropunctata* (*n* = 78), and *C. pusillus* (*n* = 58), which together accounted for 47.7% of recorded individuals in that resource. Sodium baits were primarily occupied by *C. pusillus* (*n* = 5) and *Solenopsis* sp. 2 (*n* = 4), representing 69.2% of sampled individuals in that resource. In contrast, water baits were dominated by *Azteca* sp. 1 (*n* = 16), *C. pusillus* (*n* = 13), and *Pheidole* sp. 2 (*n* = 4), comprising 89.2% of individuals at that resource. Vertically, the community showed distinct stratification: 12 species (*n* = 193) were recorded in the arboreal stratum, dominated by *C.* *pusillus* (*n* = 66), *Azteca* sp. 1 (*n* = 35), and *Pheidole* sp. 1 (*n* = 34), while 16 species (*n* = 413) were found in the epigeic stratum, led by *Pheidole sp*. 2 (*n* = 134), *W. auropunctata* (*n* = 78), and *Brachymyrmex* sp. 1 (*n* = 49). Both resource type and vegetation stratum affected ant foraging at the community level (Fig. 2; Table 1). Sugar baits attracted eight times more ant species (1.15 ± 0.17, mean ± SE) than sodium baits (0.13 ± 0.04), and seven times more than on water baits (0.16 ± 0.05), whereas species richness did not differ between sodium and water baits. Similarly, about 15 times more individuals were recruited on sugar baits (6.78 ± 1.42) than on sodium baits (0.44 ± 0.19), and 11 times more than on water baits (0.58 ± 0.23), with no difference between sodium and water. Vertically, 1.6 times more ant species (0.36 ± 0.07) and twice as many individuals (1.70 ± 0.45) recruited in the epigaeic stratum compared to the arboreal stratum (0.23 ± 0.05 and 0.85 ± 0.28, respectively). Notably, the recruitment for sugar remained consistent across both strata, as evidenced by the lack of a significant resource-stratum interaction.

**Table 1:** Generalized linear mixed model (GLMM) results evaluating overall ant community-level foraging effort (number of species and number of individuals) across different resource types (water, sodium, and sugar) and vegetation strata (ground and arboreal) in a Restinga ecosystem. The table presents Wald chi-square tests for fixed effects. Bold values indicate significant effects (*p* < 0.05). Results of Tukey-adjusted post hoc pairwise comparisons conducted using the *emmeans* package are also provided for the resource types

Response variable	Explanatory variable	Chi	DF	*p*	Pairwise comparisons
The number of ant species (community level)	Resource type	60.10	2	**< 0.001**	Sugar > Sodium (p < 0.001); Sugar > Water (p < 0.001); Sodium = Water (p = 0.8852)
	Vegetation stratum	4.46	1	**0.034**	Epigaeic > Arboreal
	Interaction	1.14	2	0.564	–
The number of ant individuals (community level)	Resource type	74.38	2	**< 0.001**	Sugar > Sodium (p < 0.001); Sugar > Water (p < 0.001); Sodium = Water (p = 0.858)
	Vegetation stratum	7.76	1	**0.005**	Epigaeic > Arboreal
	Interaction	1.17	2	0.555	–

**Fig. 3:**
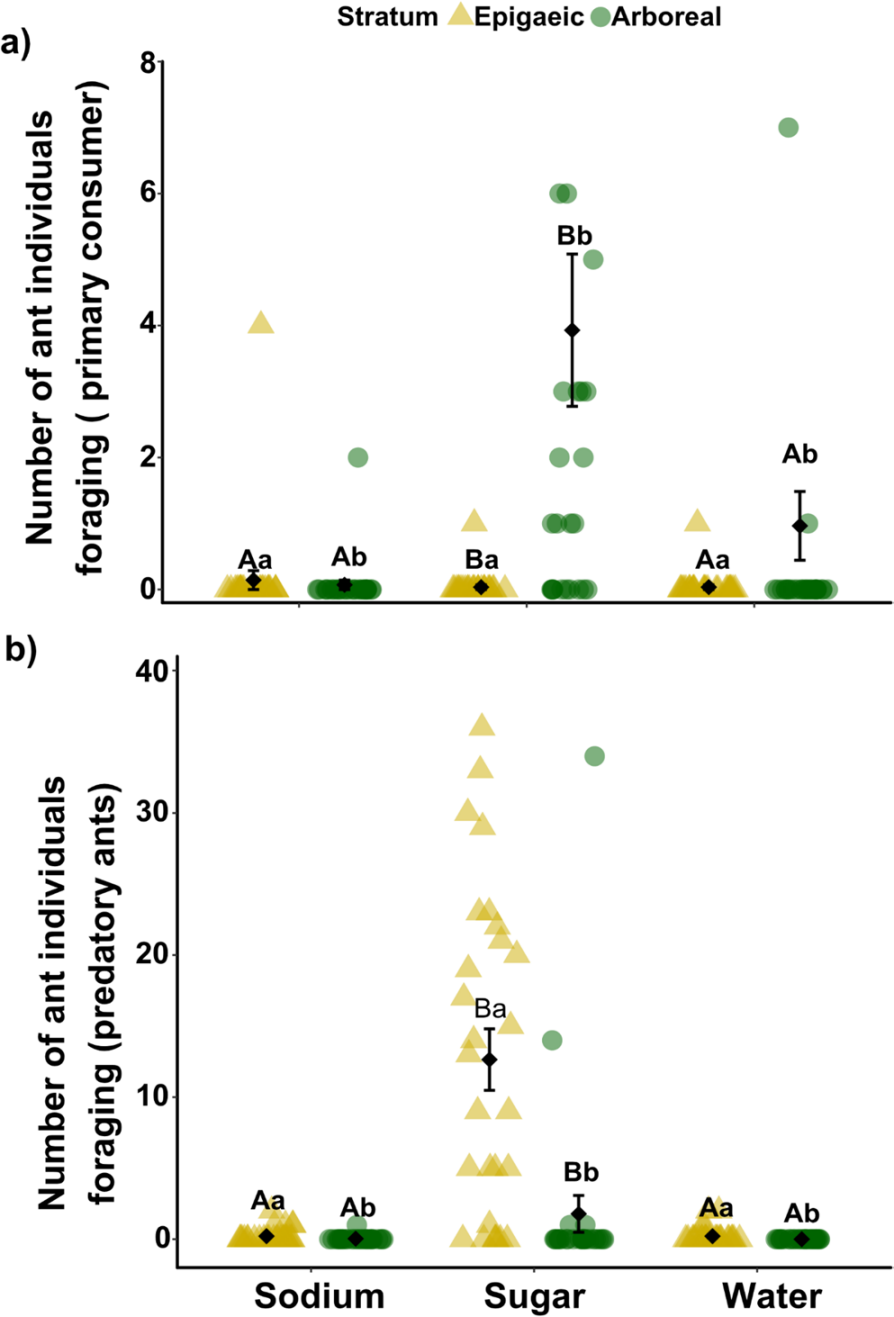
Mean and variation (SE) of the number of ants of primary consumer **(a)** and predatory ants **(b)** foraging across strata (epigaeic and arboreal) and different resources (sodium, sugar and water) within the floodable forest formation at a Brazilian Restinga (a coastal ecosystem, highly saline habitat and poor in sugar). Yellow triangles represent units in the epigeic stratum, while green circles represent units in the arboreal stratum. Black bars represent standard error, and black diamonds represent the means. Capital letters indicate post hoc comparisons among resource types, whereas small letters indicate comparisons between strata. Treatments sharing the same letter do not differ significantly, whereas different letters indicate significant differences (α = 0.05)

Disaggregating the community into feeding guilds revealed distinct spatial preferences alongside a universal attraction to carbohydrates (Fig. 3; Table 2). Predominantly primary consumers and predators both recruited strongly to sugar baits: primary consumers showed a 17-fold increase of individual ants (0.68 ± 0.30, mean ± SE) compared to sodium (0.04 ± 0.03), while predators exhibited a 42-fold increase (5.46 ± 2.32) than on sodium baits (0.04 ± 0.03) and 59 times as many as on water baits (0.09 ± 0.07). In both cases, the number of individuals recorded on water and sodium was similar. In addition, 14 times more primary consumer ants were recruited in the arboreal stratum (0.57 ± 0.24) than in the epigaeic stratum (0.04 ± 0.03), whereas 7 times more predatory ants were recruited in the epigaeic stratum (1.07 ± 0.34) than in the arboreal stratum (0.15 ± 0.13). No significant interaction was detected between resource type and vegetation stratum, for both predominantly primary consumers and predominantly predators.

**Table 2:** Generalized linear mixed model (GLMM) results evaluating ant feeding guilds’ (predominantly primary consumers and predominantly predatory ants) foraging effort (number of individuals) across different resource types (water, sodium, and sugar) and vegetation strata (ground and arboreal) in a Restinga ecosystem. The table presents Wald chi-square tests for fixed effects. Bold values indicate significant effects (*p* < 0.05). Results of Tukey-adjusted post hoc pairwise comparisons conducted using the *emmeans* package are also provided for the resource types

Response variable	Explanatory variable	Chi	DF	*p*	Pairwise comparisons
Number of individuals (predominantly primary consumer ants)	Resource type	22.85	2	**< 0.001**	Sugar > Sodium (p < 0.001); Sugar > Water (p = 0.002); Sodium = Water (p = 0.339)
	Stratum	18.34	1	**< 0.001**	Arboreal > Epigaeic
	Interaction	4.63	2	0.098	–
Number of individuals (predominantly predatory ants)	Resource type	86.63	2	**< 0.001**	Sugar > Sodium (p < 0.001); Sugar > Water (p < 0.001); Sodium = Water (p = 0.867)
	Stratum	5.83	1	**0.015**	Epigaeic > Arboreal
	Interaction	0.77	2	0.677	–

## Discussion

**Fig. 4:**
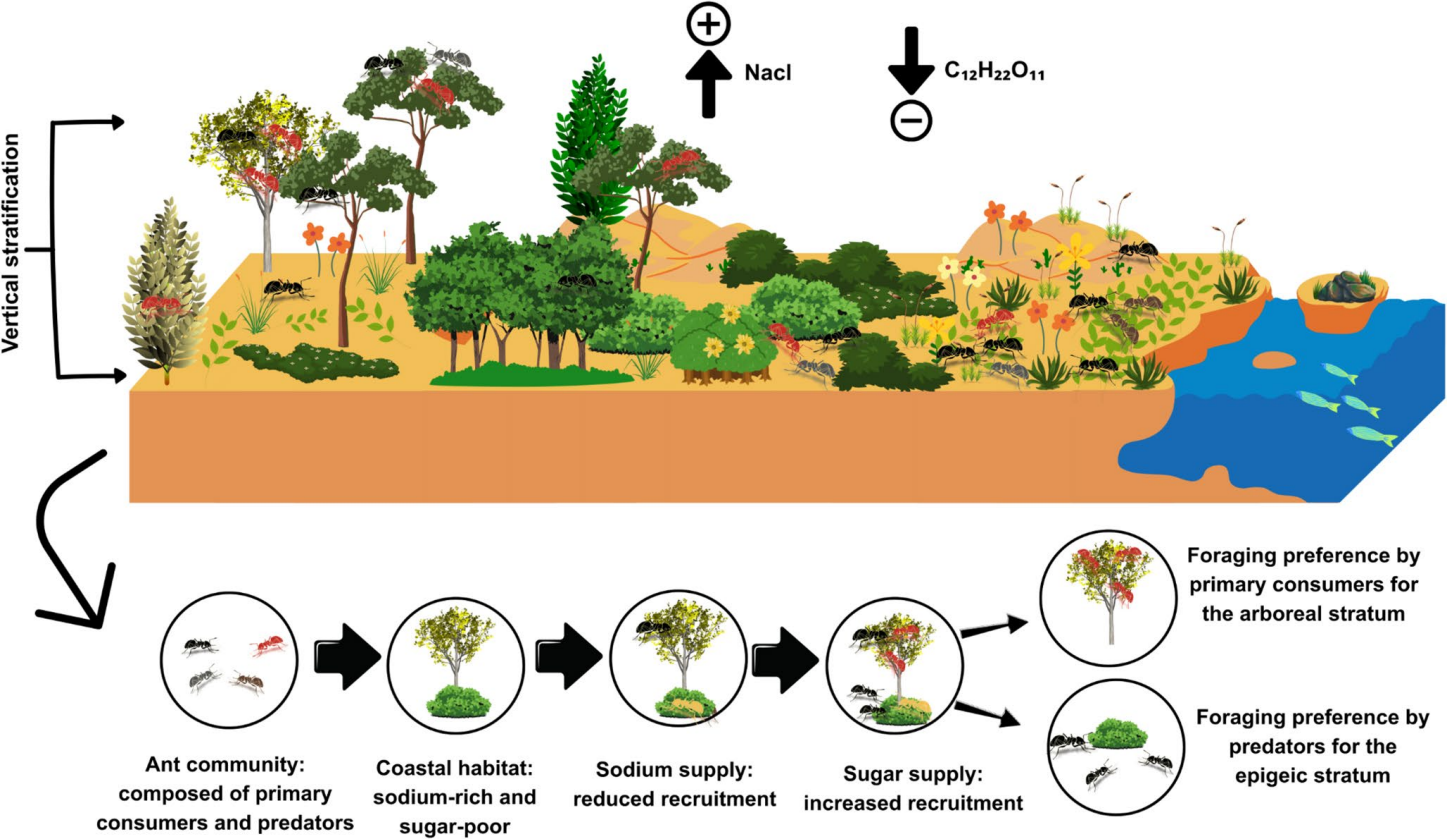
Nutritional balance searches by ants in Brazil’s Restinga, a coastal ecosystem where ants showed stronger recruitment to carbohydrate resources than to sodium resources. Like other faunal groups, ants tend to recruit more intensively for scarce resources. Despite vegetation stratification, predominantly predatory ants forage strongly in the epigeic stratum, whereas predominantly primary consumers prefer the arboreal stratum

We found that the overall ant community, including both predominantly primary consumers and predominantly predatory ants, recruits more strongly to sugar than to sodium or water, a pattern that remains consistent across all vegetation strata. This pattern is consistent with ecological stoichiometry and nutritional compensation theories, which predict that organisms intensify foraging efforts toward scarce resources to alleviate nutritional imbalances (Tilman 1982; Kay 2004; Lasmar et al. 2023). Hence, our findings indicate that carbohydrate availability may be an important driver of ant foraging behavior in saline coastal ecosystems (Fig. 4). Therefore, in these environments, resource availability can function as a critical biotic filter that modulates the overall ant community level (Cardoso and Schoereder 2014), with important implications for ecosystem functions they provide (Kaspari and Yanoviak 2009).

Consistent with our first hypothesis, we observed more recruitment at carbohydrate-based resources than at sodium- or water-based resources across the overall ant community, and both predominantly primary consumers and predominantly predatory ants. In the Brazilian Restinga, where carbohydrate-based resources are limited mainly due to the low availability of extrafloral nectaries (Miranda et al. 2022), sugar acts as a limiting resource that directs foraging behavior and likely facilitates structural shifts in the overall ant community (Belchior et al. 2016; Ribeiro et al. 2018). This drive for carbohydrates may compensate for the high energetic costs associated with foraging in high-temperature coastal ecosystems, and potentially increases competition and resource partitioning among species (Dáttilo et al. 2015; Anjos et al. 2017). Additionally, organisms in high-temperature environments, such as coastal ecosystems, experience increased energetic expenditure during foraging, which heightens their need for resources that compensate for this elevated energy demand (Stuble et al. 2013; Prather et al. 2018; Wilker et al. 2025). While we did not directly quantify soil sodium levels, the minimal interest in salt baits suggests that carbohydrates provide the strongest energetic stimulus to overcome environmental stressors across the overall ant community and both trophic guilds investigated. Predominantly primary consumer ants foraged more intensively in the arboreal stratum, supporting our second hypothesis. Coupled with their stronger recruitment to carbohydrate baits, these results indicate that primary consumer ants forage intensively for carbohydrates resources independent of their vegetation strata preference. As ants require large amounts of carbohydrates to sustain metabolism, including foraging, growth, and reproduction (Simpson and Raubenheimer 1993), carbohydrates availability in coastal ecosystems can influence their behavior and aggressiveness (Grover et al. 2007). Consequently, carbohydrate-limited environments can lead to greater ant recruitment and interspecific competition, favoring more aggressive species (Cerdá et al. 2013). Our findings indicate that even in plants lacking extrafloral nectaries, such as *Pera glabrata*, the presence of carbohydrates can trigger heightened ant activity. A similar effect was observed in *Ocotea notata*, which also lacks extrafloral nectaries: carbohydrates supplementation increased ant-mediated plant defenses (Terra et al. in preparation). This suggests that the artificial or natural pulse of carbohydrates in the vegetation of coastal ecosystems can enhance vital ecosystem services, such as ant-mediated plant defense, by increasing the local density and activity of predominantly primary consumer ants.

Predominantly predatory ants foraged more strongly in the epigeic than in the arboreal stratum, and, like predominantly primary consumers, they recruited more strongly to carbohydrate baits in both strata, supporting our third hypothesis. While predominantly predatory ants require protein from prey and seeds for colony growth, larval development and reproduction, our results highlight their significant dependency on carbohydrates to fuel adult foraging activity. In the carbohydrate-poor Restinga, predatory ants appear to forage across vegetation strata to compensate for this deficit. Hence, as predatory ants can feed on extrafloral nectar and seeds, they expand their ecological roles, potentially serving as both seed dispersers and protectors of plant against insect herbivores (Passos and Oliveira 2003; Cogni and Oliveira 2004). These findings, therefore, align with those observed for primary consumers: as carbohydrates can trigger increased ant recruitment, especially in habitats where it is scarce (Cerdá et al. 2013), an artificial or natural pulse of carbohydrates across both strata may drive a vertical distribution of foraging effort toward this resource, particularly among the more active species. However, unlike predominantly primary consumers, predominantly predatory ants recruited more strongly in the epigeic stratum, which may enhance their functional roles within this stratum. For instance, species of the omnivorous genus *Pheidole*, known for their activity and aggressiveness (Campos et al. 2011), were dominant in this study, with six species exhibiting intense recruitment (Table S3-S4). This dominance suggests that carbohydrates recruitment is shaped by both nutritional needs and competitive interactions. Dominant ants can monopolize high-quality sugar resources, potentially masking even higher latent demand from subordinate species. Ultimately, our data suggest that despite the foraging vertical stratification, carbohydrates availability acts as a key resource that homogenizes the foraging priorities of both predominantly predators and predominantly primary consumers. Nutrient distribution in Restinga ecosystems is shaped by environmental factors such as vegetation structure, plant species composition, soil nutrient availability and microclimatic conditions (Cardoso and Schoereder 2014). Because carbohydrates resources are largely derived from plants and honeydew-producing insects, spatial variation in vegetation likely creates a mosaic of nutritional opportunities (Ribas et al. 2003). Anthropogenic pressures such as habitat loss, fragmentation and vegetation simplification may further reduce the abundance and spatial distribution of these resources, potentially altering ant foraging dynamics and their ecological interactions (Dátillo and MacGregor-Fors 2021; Sá et al. 2026). While our study focused on a single floodable forest formation, fine-scale salinity gradients along the coast–inland transition may further shift these nutritional priorities. Future studies combining resource supplementation experiments with direct measurements of soil and plant tissues would help clarify how spatial variation in salinity influences ant resource use in coastal ecosystems.

In conclusion, our findings indicate that experimentally supplied resources can strongly influence ant foraging patterns across trophic guilds and vegetation strata in coastal Restinga ecosystems. Hence, we expand our knowledge about how the interplay between resource abundance and scarcity can shape biological communities across natural systems. Because ants are extremely abundant and engage in several ecological interactions, their intense prioritization of carbohydrates in this coastal ecosystem likely triggers cascading effects on plant-herbivore interactions, seed dynamics, and trophic regulation. Thus, resource scarcity is a crucial factor modulating foraging strategies across habitat strata and trophic groups in the Restinga. Consequently, environmental changes that further deplete carbohydrate resources – whether through climate change or habitat degradation – may significantly alter ant foraging behaviour, with broader consequences for coastal ecosystems functioning (Houadria and Menzel 2017). Because ants are key ecological actors in these environments, changes in the availability of carbohydrate-rich resources may have broader consequences for species interactions and ecosystem functioning.

## Supplementary Information

Below is the link to the electronic supplementary material.

Supplementary Material 1 (PDF 211 KB)

## Data Availability

The data used in this study are available upon reasonable request.
