# Supplementary material for: Carbohydrate resources drive increased ant recruitment in a saline coastal ecosystem
Source: Naturwissenschaften. 2026 Jul 20;113(4):87. doi: 10.1007/s00114-026-02136-w (PMC13385417; doi:10.1007/s00114-026-02136-w)
Supplement: Supplementary file 1 — Supplementary Material 1 (PDF 211 KB) [file 114_2026_2136_MOESM1_ESM.pdf]

Carbohydrate resources drive increased ant recruitment in a saline coastal ecosystem

Table S1 Ant species sampled in June 2023 in the epigeic and arboreal strata of the flooded forest formation at Parque Estadual de Itaúnas, ES, and their respective trophic levels according to feeding habits.

Subfamily	Ant species	Primary consumers	Predators	Categorization Method
Dolichoderinae	*Azteca* sp.1	X		Field observation
Ectatomminae	*Ectatomma edentatum*		X	Literature
	Ectatomma planidens		X	Literature
	Ectatomma tuberculatum	X		Field observation
Formicinae	*Brachymyrmex* sp.1	X		Literature
	Brachymyrmex sp.2	X		Literature
	Camponotus crassus		X	Literature
	Camponotus rectangularis		X	Field observation
	Camponotus sp.1		X	Literature
	Nylanderia sp.1	X		Literature
	Cephalotes pusillus	X		Literature
	Crematogaster sp.1		X	Literature

Myrmicinae	*Monomorium floricola*	X		Literature
	Pheidole sp.1		X	Literature
	Pheidole sp.2		X	Literature
	Pheidole sp.3		X	Literature
	Pheidole sp.4		X	Literature
	Pheidole sp.5		X	Literature
	Pheidole sp.6		X	Literature
	Solenopsis sp.1		X	Literature
	Solenopsis sp.2		X	Literature
	Wasmannia auropunctata		X	Literature
Ponerinae	*Neoponera villosa*		X	Literature
Pseudomyrmecinae	*Pseudomyrmex* sp.1	X		Literature
	Pseudomyrmex sp.2	X		Literature

Table S2 Descriptive data for variables measured: minimum, maximum, mean, and standard deviation value of response variables (the number of individuals and species at community level and the number of ant individuals of primary consumer ants and predatory ants) across resource supplementation and stratum (sugar, sodium and water / epigaeic and arboreal).

	**Resource/Strata**	**Min–Max**	**Mean ± DP**
**Ant community richness**	Sugar	0 – 5	1.25 ± 1.11
	Sodium	0 – 2	0.14 ± 0.40
	Water	0 – 2	0.17 ± 0.43
	Epigaeic stratum	0 – 5	0.64 ± 1.04
	Arboreal stratum	0 – 2	0.40 ± 0.67
**Ant community abundance**	Sugar	0 – 36	9.19 ± 10.62
	Sodium	0 – 5	0.23 ± 0.78
	Water	0 – 10	0.60 ± 2.00
	Epigaeic stratum	0 – 36	4.42 ± 8.81
	Arboreal stratum	0 – 35	2.26 ± 5.69
**Abundance of primary consumers ants**	Sugar	0 – 28	1.98 ± 4.70
	Sodium	0 – 4	0.10 ± 0.59
	Water	0 – 10	0.50 ± 1.99
	Epigaeic stratum	0 – 4	0.07 ± 0.46
	Arboreal stratum	0 – 28	1.65 ± 4.16
**Abundance of predatory ants**	Sugar	0 – 36	7.21 ± 10.83
	Sodium	0 – 2	0.12 ± 0.38
	Water	0 – 2	0.10 ± 0.41
	Epigaeic stratum	0 – 36	4.35 ± 8.80
	Arboreal stratum	0 – 34	0.60 ± 3.39

Table S3 The number of ant individuals (recruitment) by species across supplemented resources

**Ant species**	**sodium**	**sugar**	**water**	**Total**
Azteca sp1	0	19	16	35
Brachymyrmex sp1	1	48	0	49
Brachymyrmex sp2	0	4	0	4
Camponotus crassus	0	1	0	1
Camponotus rectangularis	0	1	0	1
Camponotus sp1	0	1	0	1
Cephalotes pusillus	5	58	13	76
Crematogaster sp1	0	16	0	16
Ectatomma edentatum	0	7	0	7
Ectatomma planidens	0	4	0	4
Ectatomma tuberculatum	0	4	0	4
Monomorium floricola	0	28	0	28
Neoponera villosa	0	1	0	1
Nylanderia sp1	0	2	0	2
Pheidole sp1	0	34	0	34
Pheidole sp2	1	129	4	134
Pheidole sp3	1	2	0	3
Pheidole sp4	0	0	1	1
Pheidole sp5	0	44	0	44
Pheidole sp6	0	43	0	43
Pseudomyrmex sp1	1	0	2	3
Pseudomyrmex sp2	0	1	0	1
Solenopsis sp1	0	31	1	32
Solenopsis sp2	3	0	0	3
Solenopsis sp22	1	0	0	1
Wasmannia auropunctata	0	78	0	78

Table S4 The number of ant individuals (recruitment) by species across strata

Ant species	epigeic	arboreal	Total
Azteca sp1	0	35	35
Brachymyrmex sp1	49	0	49
Brachymyrmex sp2	0	4	4
Camponotus crassus	1	0	1
Camponotus rectangularis	0	1	1
Cephalotes pusillus	10	66	76
Crematogaster sp1	0	16	16
Ectatomma edentatum	7	0	7
Ectatomma planidens	4	0	4
Ectatomma tuberculatum	0	4	4
Monomorium floricola	1	27	28
Neoponera villosa	0	1	1
Nylanderia sp1	2	0	2
Pheidole sp1	0	34	34
Pheidole sp2	134	0	134
Pheidole sp3	3	0	3
Pheidole sp4	1	0	1
Pheidole sp5	44	0	44
Pheidole sp6	43	0	43
Pseudomyrmex sp1	0	3	3
Pseudomyrmex sp2	0	1	1
Solenopsis sp1	32	0	32
Solenopsis sp2	3	0	3
Solenopsis sp22	0	1	1
Wasmannia auropunctata	78	0	78
Camponotus sp1	1	0	1
